# 1-deoxy-D-xylulose-5-phosphate synthase from *Pseudomonas aeruginosa* and *Klebsiella pneumoniae* reveals conformational changes upon cofactor binding

**DOI:** 10.1016/j.jbc.2023.105152

**Published:** 2023-08-09

**Authors:** Rawia Hamid, Sebastian Adam, Antoine Lacour, Leticia Monjas, Jesko Köhnke, Anna K.H. Hirsch

**Affiliations:** 1 Department of Drug Design and Optimization, Helmholtz Institute for Pharmaceutical Research Saarland (HIPS) – Helmholtz Centre for Infection Research (HZI), Saarbrücken, Germany; 2 Department of Pharmacy, Saarland University, Saarbrücken, Germany; 3 Stratingh Institute for Chemistry, University of Groningen, Groningen, The Netherlands; 4 Institute of Food Chemistry, Leibniz University Hannover, Hannover, Germany; 5 School of Chemistry, University of Glasgow, Glasgow, UK

**Keywords:** 1-deoxy-D-xylulose 5-phosphate synthase, *Pseudomonas aeruginosa*, X-ray crystallography, DXPS, conformational changes, *Klebsiella pneumonia*

## Abstract

The ESKAPE bacteria are the six highly virulent and antibiotic-resistant pathogens that require the most urgent attention for the development of novel antibiotics. Detailed knowledge of target proteins specific to bacteria is essential to develop novel treatment options. The methylerythritol-phosphate (MEP) pathway, which is absent in humans, represents a potentially valuable target for the development of novel antibiotics. Within the MEP pathway, the enzyme 1-deoxy-D-xylulose-5-phosphate synthase (DXPS) catalyzes a crucial, rate-limiting first step and a branch point in the biosynthesis of the vitamins B1 and B6. We report the high-resolution crystal structures of DXPS from the important ESKAPE pathogens *Pseudomonas aeruginosa* and *Klebsiella pneumoniae* in both the co-factor-bound and the apo forms. We demonstrate that the absence of the cofactor thiamine diphosphate results in conformational changes that lead to disordered loops close to the active site that might be important for the design of potent DXPS inhibitors. Collectively, our results provide important structural details that aid in the assessment of DXPS as a potential target in the ongoing efforts to combat antibiotic resistance.

The widespread use of antibiotics over the past five decades has caused bacteria to develop resistance mechanisms to evade the detrimental effect of these agents (1, 2). This effect is especially critical for infections caused by ESKAPE pathogens, which are difficult to treat and result in increasing fatality rates. *Pseudomonas aeruginosa and Klebsiella pneumoniae* are both Gram-negative pathogens with the ability to integrate exogenous DNA to obtain antibiotic resistance (horizontal gene transfer) (3). Both organisms can adapt to the environment of human airways (4) and are major causes of opportunistic infections, predominantly, pneumonia and sepsis in hospitalized patients and patients with cystic fibrosis (5, 6). Measures such as the antibiotic stewardship program to improve the way antibiotics are prescribed, and the synthesis of novel chemical entities with new modes of action need to be implemented to tackle the antimicrobial-resistance crisis.

The methylerythritol-phosphate (MEP) pathway has attracted attention as it represents a rich source of potentially attractive anti-infective drug targets. The MEP pathway, also called the mevalonate-independent pathway, is considered to be the main pathway for the synthesis of isoprenoid building blocks in most bacteria, plants, and protozoa (7, 8, 9). Its absence in humans makes the enzymes of this pathway, particularly interesting targets for anti-infective drug discovery. The complete pathway consists of seven enzymes, each of which could be targeted individually. 1-Deoxy-D-xylulose-5-phosphate synthase (DXPS) catalyzes the first rate-limiting step of the MEP pathway. The enzyme is responsible for the formation of 1-deoxy-D-xylulose 5-phosphate (DXP) by condensation of pyruvate and glyceraldehyde 3-phosphate (D-GAP) in the presence of thiamine diphosphate (ThDP), Figure 1. DXP, in and of itself, is an important metabolite in not only the biosynthesis of isoprenoid precursors but also in vitamin B1 (thiamine) and vitamin B6 (pyridoxine) biosynthesis (10). DXPS is a promising anti-infective drug target (11, 12, 13).

**Figure 1:**
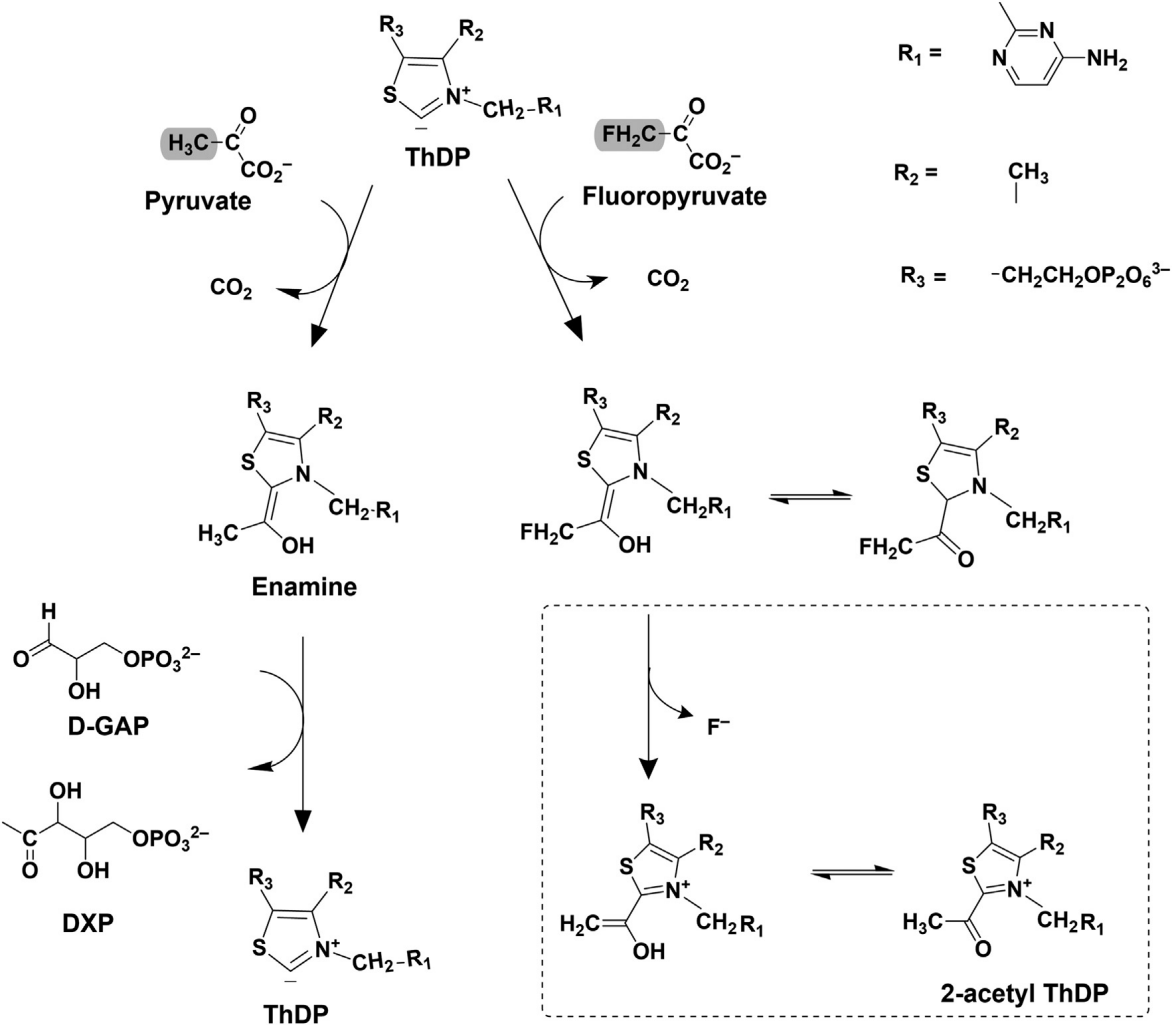
**DXPS reaction mechanism with pyruvate and fluoropyruvate.** Suggested formation of the fluoropyruvate adduct (2-acetyl ThDP) catalyzed by *pa*DXPS in combination with ThDP in comparison to the reported reaction with the natural substrate pyruvate. The point of difference between fluoropyruvate and pyruvate is highlighted in *gray*.

The first DXPS structures of *Deinococcus radiodurans* and *Escherichia coli* were solved *via* X-ray crystallography and published in 2007 (14). Subsequent studies on these structures have shown that DXPS undergoes a number of conformational changes during catalysis which takes place near the substrate-entry site (15, 16, 17). The ThDP-bound enzyme, in the presence of pyruvate, forms a pre-decarboxylation intermediate, lactyl thiamine diphosphate (LThDP) (15, 18). The enzyme adopts a closed conformation when bound to LThDP and exposure to D-GAP or DXP triggers a conformational change to an open conformation, which seemingly initiates LThDP decarboxylation (17, 19). Chen *et al.* coined the name spoon-fork motif to describe the shape of the region that shows conformational changes during catalysis. They have shown that binding of LThDP results in a closed conformation of the spoon motif that opens when the subsequent intermediate enamine is formed (17). These results highlight the important role of conformational changes in DXPS catalysis. Further investigation is needed to expand our knowledge about the dynamic catalysis and structural differences of this unique enzyme.

Given that the binding site of the cofactor ThDP is the most promising site for drugs targeting ThDP-dependent enzymes including DXPS (16, 20, 21), it is also vital to understand the precise location of ThDP and the general three-dimensional shape of the pocket. It is also considered the main druggable pocket in DXPS enzymes (22). Thus far, few experimentally determined structures of DXPS have been reported (23), but due to the generally conserved active sites, the available crystal structures of DXPS are used as models for structure-based drug design (16, 20, 21). However, this has proven to be very limiting, as three-dimensional active sites can typically not be deduced from sequence homology alone. To improve our understanding of DXPS active-site architecture and to provide a better framework for drug-discovery efforts addressing this promising target, we determined the crystal structures of both the *apo* and the cofactor-bound forms from the ESKAPE pathogens *P. aeruginosa* and *K. pneumoniae*. We demonstrate that the binding of ThDP leads to conformational changes involving a loop in domain I, which alters the ThDP biding site and contributes to the binding of the ThDP diphosphate. Together with the structure of *P. aeruginosa* DXPS bound to the less reactive 2-acetylThDP, we provide a firm basis for future drug design and -discovery efforts targeting this important enzyme from the MEP pathway (2).

## Results and discussion

### Expression, purification, and kinetic characterization of *DXPS*

Our group recently reported a truncated *D. radiodurans DXPS* (*dr*DXPS) construct in which a flexible loop has been modified to produce a protein that crystallizes readily and yields high-quality crystals that diffract to a resolution better than 2.0 Å (24). This loop typically has very low evolutionary conservation and neither shapes the active site nor is it involved in the formation of a secondary structure (24). Our construct served as a blueprint for the alteration in DXPS enzymes from different organisms (23). After multiple sequence alignments of published sequences, we identified the loop to be corresponding to residues 206 to 245 in *pa*DXPS and residues 197 to 240 in *kp*DXPS (Fig. S1) and replaced them with a six glycine unit. We then heterologously expressed the enzymes in *E. coli*, purified and analyzed them as detailed in the Experimental procedures section. Next, we analyzed the activity of new homolog constructs, evaluated, and compared the enzyme kinetics for both versions of the enzyme *i.e.* truncated and full-length, of the enzyme. The results summarized in Table 1 demonstrate comparable activities of the native and truncated *pa*DXPS, both versions of the enzyme show similar affinities for the substrate and co-factor. Although native *kp*DXPS was not tested, the truncated version is showing comparable kinetics for both substrates. No kinetics values for ThDP were reported for other homologs, which makes our data impossible to compare to literature values. However, *kp*DXPS shows a slightly higher affinity for the substrates and elevated catalytic activity in comparison to *pa*DXPS, which may be due to the different shape of the binding site. The kinetic parameters remain within a similar range for *pa*DXPSs, and the preserved enzymatic activity of *kp*DXPS suggests that the truncation does not affect any catalytically significant residues. These results allowed us to use these optimized and more stable constructs for biological testing as well as structure determination. After confirming the identity and activity of our purified enzymes, we opted to elucidate the structures of these novel homologs from ESKAPE pathogens.

**Table 1:** The steady-state kinetics parameters of ThDP, pyruvate, and D-GAP for the wild-type and mutated *pa*DXPS and mutated *kp*DXPS

Enzyme	Native *pa*DXPS 0.15 μmol/L	Truncated *pa*DXPS 0.15 μmol/L	Truncated *kp*DXPS 0.20 μmol/L
Pyruvate	*K*_*m*_: 184 ± 25 μM *K*_*cat*_: 1.6 ± 0.01 × 10^3^ min^−1^	*K*_*m*_: 122 ± 8.2 μM *K*_*cat*_: 1.2 ± 0.02 × 10^3^ min^−1^	*K*_*m*_: 113 ± 34 μM *K*_*cat*_: 2.1 ± 0.03 × 10^3^ min^−1^
D-GAP	*K*_*m*_: 357 ± 77 μM *K*_*cat*_: 1.8 ± 0.02 ×10^3^ min^−1^	*K*_*m*_: 336 ± 98.0 *K*_*cat*_: 1.5 ± 0.03 × 10^3^ min^−1^	*K*_*m*_: 170 ± 43 μM *K*_*cat*_: 2.4 ± 0.01 × 10^3^ min^−1^
ThDP	*K*_*m*_: 70 ± 9.0 nM *K*_*cat*_: 1.6 ± 0.01 × 10^3^ min^−1^	*K*_*m*_: 96.5 ± 14 nM *K*_*cat*_: 0.94 ± 0.01 × 10^3^ min^−1^	*K*_*m*_: 110 ± 17.0 nM *K*_*cat*_: 2.6 ± 0.03 × 10^3^ min^−1^

When measuring *K*_m_, the other substrate and/or cofactor concentrations were kept in excess to ensure the enzyme reaches maximum velocity.

### Structure determination of DXPS from *K. pneumoniae and P. aeruginosa*

The optimized constructs of DXPS from *K. pneumoniae* and *P. aeruginosa* were used for crystallization trials. The structures were determined using molecular replacement with the published DXPS structure of *E. coli* (PDB ID: 2o1s) as a search model. The two proteins in the asymmetric unit form the expected biological dimer known from other DXPS enzymes, which was supported by native MS measurements for *pa*DXPS (Fig. S2). The low solubility of *kp*DXPS precluded native MS data acquisition, but an analysis of the dimer observed in the crystal structure using the PISA server (25) and analytical gel filtration (Fig. S3) confirmed a stable dimer interface, which is highly similar between all DXPS structures. Although we determined a comparable affinity for ThDP, we can only observe electron density for ThDP in one of the two protomers of the dimer of *kp*DXPS with low occupancy. This may be the result of the longer crystal formation time of 20 days and the consequent degradation of the cofactor. Intriguingly, the N-terminus of this protomer extends into the cofactor binding site of the dimer partner up to Ser186. Given that this would lead to an inactive enzyme and the absence of this arrangement from the apo structures (see below), we consider this arrangement a crystallographic artifact representing a low-energy state of this particular crystal form. The addition of ThDP was essential to obtain this crystal form, and we thus screened new conditions to obtain an apo structure. Crystals of *apo kp*DXPS were obtained at a considerably higher concentration than the cofactor-bound version and crystals grew more quickly. They belonged to space group C 222_1_ and a complete 2.1 Å dataset was collected at ESRF beamline ID23 to 1. The structure was solved using molecular replacement with a refined ThDP-bound *kp*DXPS structure as the search model.

Similar to *kp*DXPS, we chose an optimized construct to obtain a crystal structure of DXPS from *P. aeruginosa (pa*DXPS). The structure was determined to 2.0 Å with molecular replacement using the published structure of *dr*DXPS (PDB ID: 6OUV) as a search model. Subsequently, a refined *pa*DXPS structure was used as the search model for the molecular replacement of structures of ThDP-bound *pa*DXPS (2.3 Å), a structure with 2-acetylThDP (2.0 Å), and a co-crystal structure with a selected ThDP analog (2.2 Å). In general, paDXPS crystallized in orthorhombic crystal form, space group P2_1_2_1_2_1_. The asymmetric unit of all *pa*DXPS structures consisted of six protomers, four of which formed dimers with proteins within the asymmetric unit, while the remaining two protomers formed dimers with symmetry mates. In most obtained *pa*DXPS structures, approximately the first 30 N-terminal residues are disordered. Native MS data on *pa*DXPS confirmed that the enzyme exists as a dimer in solution (Fig. S2), which is consistent with the crystallographic results and suggests that the dimer is the biological unit *in vivo*. All data collection and refinement statistics can be found in Table S2, and the structures were deposited in the PDB.

### Structural comparison of *kp*DXPS and *pa*DXPS

**Figure 2:**
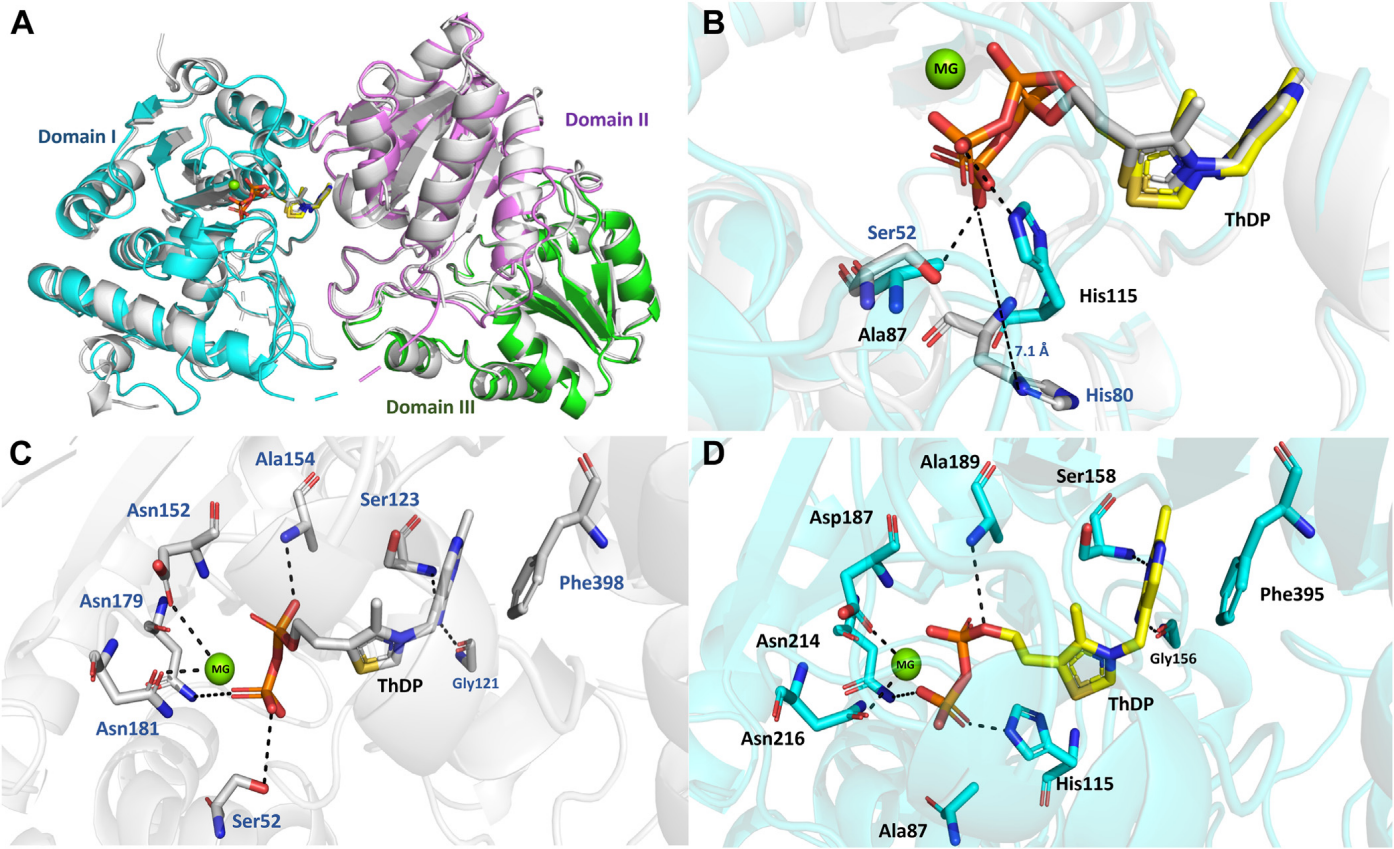
**Overview of the overall structures of *pa*DXPS and *kp*DXPS.***A*, domain organization; domain I (*cyan*; residues 1–306) contains a five-stranded parallel β-sheet, domain II (*violet*; residues 317–493) contains a six-stranded parallel β-sheet, domain III (*green*; residues 496–620) contains a five-stranded β-sheet, in *pa*DXPS, aligned with *kp*DXPS structure in gray. *B*, comparison of ThDP-bound *kp*DXPS and *pa*DXPS structures highlighting differences in the active site. *pa*DXPS is shown in *cyan*, overlaid with *kp*DXPS in *gray*, different residues from *kp*DXPS are labeled in *blue*. *C* and *D*, important interactions with ThDP in *pa*DXPS and *kp*DXPS, respectively. Interactions are shown as *black*, *dashed lines* (below 5 Å). All figures of this type were created in PyMol.

*kp*DXPS and *pa*DXPS share high sequence homology with previously identified homologs from *E. coli* (*ec*DXPS) and *D. radiodurans* (*dr*DXPS) and *Mycobacterium tuberculosis* (*mt*DXPS) (14, 23). *kp*DXPS and *pa*DXPS share 62.5% sequence identity and 84.3% sequence similarity. Consequently, the structures are also highly similar with a C_α_-RMSD of only 0.716 when comparing both ThDP-bound crystal structures. Each DXPS monomer in all structures we determined consists of three domains. The domain organization is shown in Figure 2*A* and is highly similar to the other homologs. The loop connecting domains I and II (residues 307–315 in *pa*DXPS) is highly flexible and appears to be disordered in most structures. Similar to other reported homologs, the binding site of ThDP is located at the interface between domains I and II with the pyrimidine ring facing domain II and the diphosphate moiety pointing toward domain I. ThDP adopts a chair-like conformation; the pyrimidine ring is perpendicular to the thiazole ring, which is, in turn, perpendicular to the diphosphate. In the *pa*DXPS structure, the pyrimidine ring of ThDP is π-π stacked against Phe395, while N3 of the pyrimidine is forming a backbone hydrogen bond (2.9 Å) with Ser158. Residues His115, Asp187, Ala189, Asn214, Asn216, and Lys286 are coordinating the diphosphate/Mg^2+^ part of ThDP (Fig. 2*D*). The active site of *kp*DXPS shows an almost identical binding mode of ThDP, and similar residues are involved in binding. The diphosphate is coordinated in an asparagine-rich region, with the contribution of Ser52, which forms an H bond with the diphosphate. This residue is an alanine in the *pa*DXPS structure. Among other structurally characterized DXPS homologs, *pa*DXPS, together with *mt*DXPS, seem to be the only homologs in which this hydrogen bond cannot be formed. In *mt*DXPS, this residue is changed to proline (Fig. S4). Further comparison of the two DXPS structures, reveals a key difference in the active site. His115, in *pa*DXPS, is positioned to form a hydrogen bond with the diphosphate of ThDP (Fig. 2*B*). This interaction cannot be formed in *kp*DXPS as the histidine side chain is now 7 Å away from the diphosphate (Fig. 2*B*). This may be caused by Ser52 preventing His80 from adopting the conformation seen in *pa*DXPS (steric hindrance). The H bonding interaction with His115 specific to *pa*DXPS seems to be compensating for the loss of the interaction with diphosphate caused by the change from serine to alanine in position 87 (Fig. 2*D*). This residue might be an important distinction for drug design, as the hydroxyl group of serine adds a targetable nucleophilic moiety to the active site. Fig. S4 shows the active site differences between the structures of the published DXPS homologs from *E. coli*, *D. radiodurans*, and *M. tuberculosis* and our structures. Of these, the most noteworthy is the change of Ala189 placed to make direct backbone interaction with the N2 of the pyrimidine in the homolog from the non-pathogenic bacterium *D. radiodurans* to serine (Ser158 in *pa*DXPS and Ser123 in *kp*DXPS, Fig. 2, *C* and *D*) as well as the homologs from the pathogenic *E. coli*, where the hydroxyl group of the serine side chain can again be targeted by hydrogen bonding with a ligand.

### Structural analysis of the conformational difference in the binding site between ThDP-bound and *apo* DXPS

**Figure 3:**
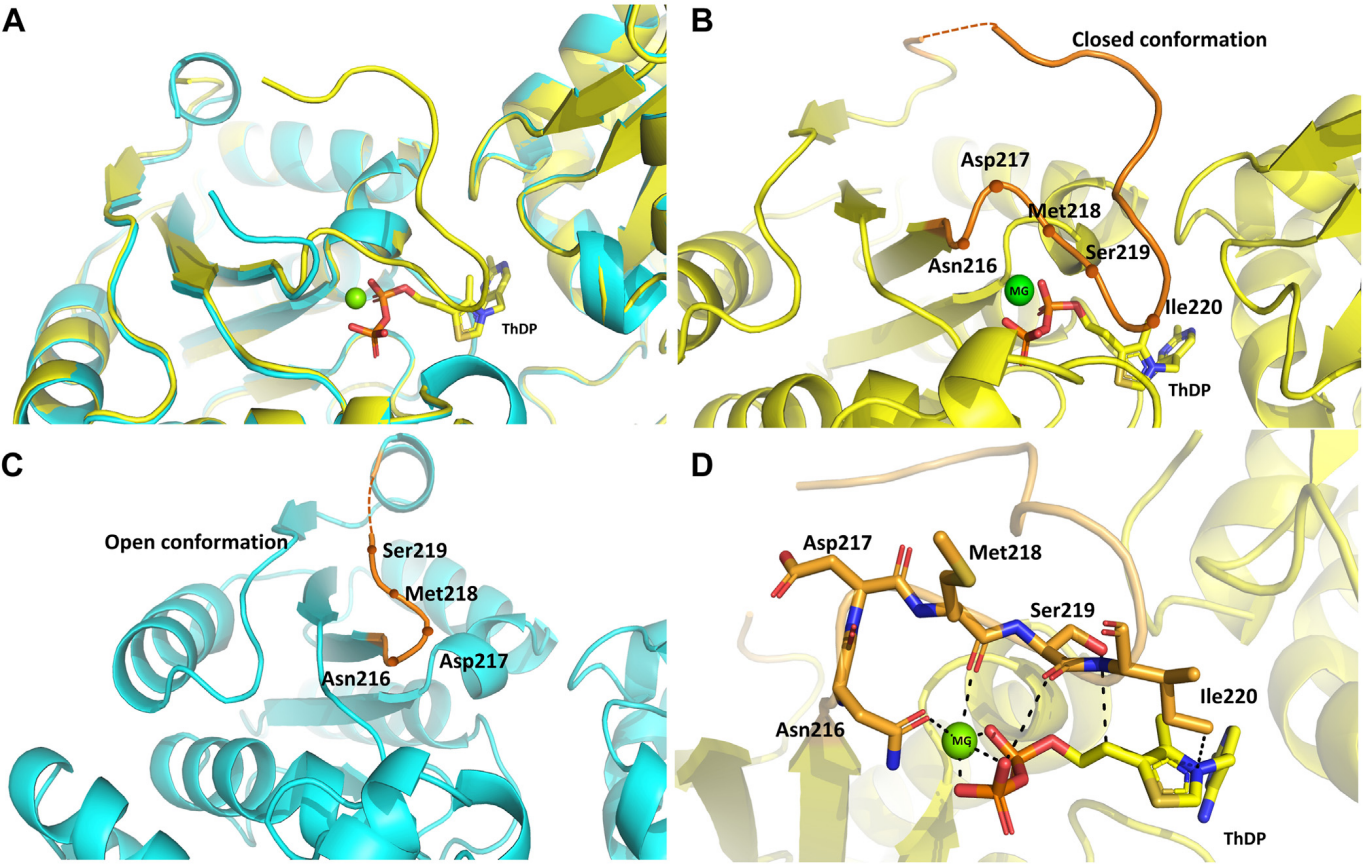
**Diphosphate loop movement in in the active site of *pa*DXPS.***A*, structure of ThDP-bound *pa*DXPS in yellow is superimposed with the structure of *apo pa*DXPS in cyan, showing the conformational change of the active site in the bound and unbound state. *B*, loop in the ThDP bound structure extending from Asn216 to Trp250, in *orange* exhibiting a closed conformation; *C*, the open conformation of the same loop. *D*, highlighting residues, Asn216, Asp217, and Met218, involved in the coordination with the diphosphate. Interactions are shown as *black*, *dashed lines* (below 5 Å). All figures of this type were created in PyMol.

**Figure 4:**
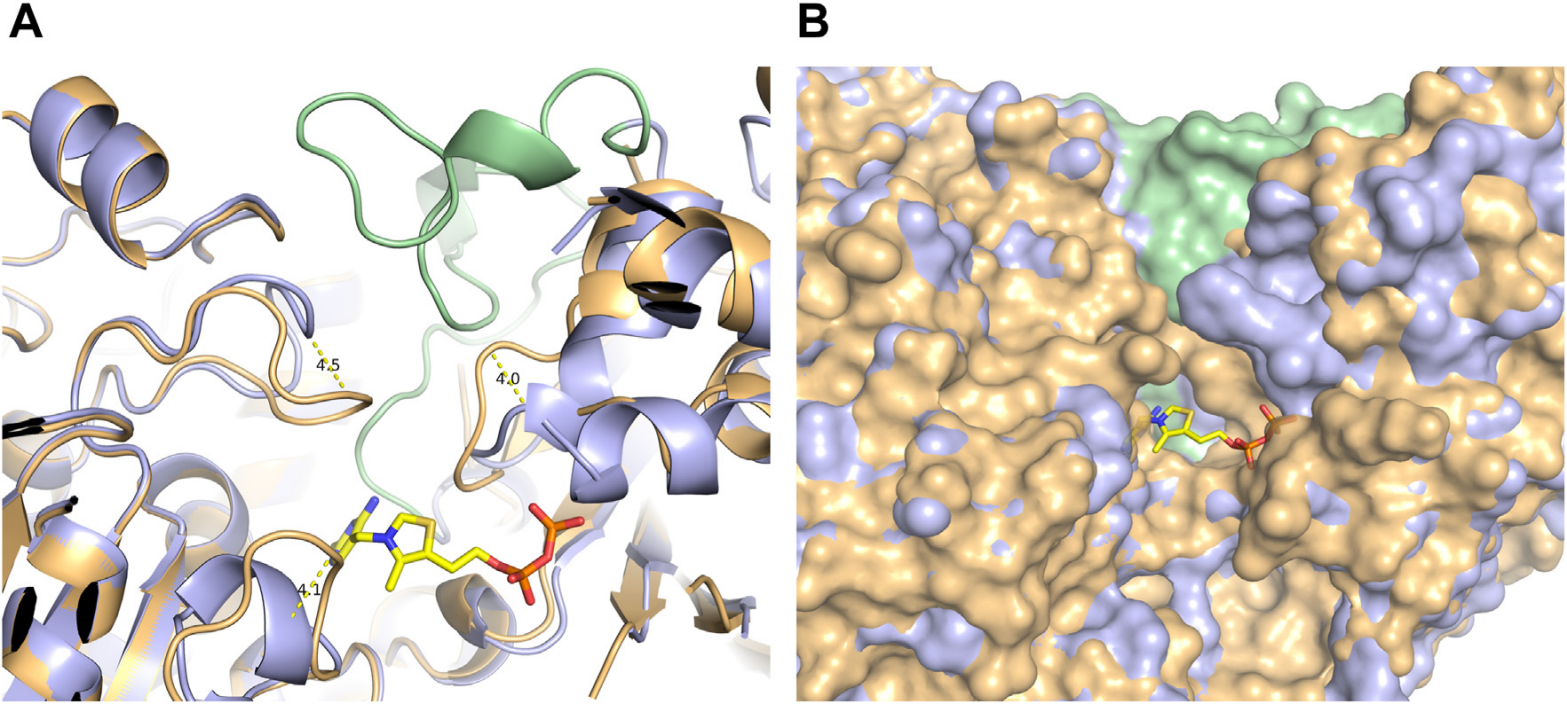
**Loops rearrangement close to the active site in *kp*DXPS.***A*, the two versions of *kp*DXPS are visualized as a cartoon representation in *light blue* (ThDP-bound) and *light orange* (*apo*). An N-terminal disordered loop in *apo kp*DXPS (amino acids 91–124) is shown in *pale green*. *B*, comparison of the surface representation of the *kp*DXPS active site, with the same color-coding as in (*A*). Loop movement leads to a much narrower pocket in *apo kp*DXPS, however, the missing N-terminal loop opens up a tunnel on the other side of the cofactor-binding site.

The influence of cofactor binding on the shape of the active site was evaluated by obtaining structural information on the *apo pa*DXPS and *kp*DXPS and comparing it to the cofactor-bound proteins. The loop spanning from Asn216 to Trp250, contributing to the ThDP binding site, exhibits a conformational change between the *holo* and the *apo* structure of *pa*DXPS (Fig. 3*A*). In the cofactor-bound version, residues As217, Met218, Ser219, and Ile220 fold around the diphosphate moiety (Fig. 3). The coordination of the co-factor bound Mg^2+^ by the side chain of Asn216 and the main chain of Met218 appear to stabilize the loop. The ThDP-bound structure also shows the participation of the ser219 and Ile220 side chains, with the latter showing the potential to form an arene-H interaction with the thiazole ring (Fig. 3*D*). Most other DXPS structures exhibit disorder in this particular region. Furthermore, our study presents the first report of an *apo* DXPS structure, making it challenging to directly compare our results with those of other homologs. In the *kp*DXPS structure regions near the active site seem to undergo various conformational changes. We observe loop rearrangement around the active site: Three loops are partially occluding the active site in the apo structure, and appear to get displaced by the bound co-factor, resulting in larger movements averaging around 4 Å (Fig. 4). The loop belonging to the domain I of DXPS (amino acids 91–124) is completely disordered in the *apo* structure of *kp*DXPS, leading to a more open conformation towards the other side of the substrate entrance channel (pale green region in (Fig. 4, *A* and *B*). Unfortunately, this part of the protein could not be resolved sufficiently to allow us to compare it to the structure of *pa*DXPS. Furthermore, while the structure obtained for the *apo kp*DXPS has a Rfree value of 20%, indicating a reasonable fit to the experimental data, it is important to state that the Wilson B factor is relatively high at 35 Å^2^. This could be linked to the disordered regions observed in the absence of the cofactor, showcasing the role of ThDP in stabilizing the overall structure of DXPS. The distinct behavior of the loops surrounding the active sites of the two homologs may have important implications for inhibitor design, as a different three-dimensional fold needs to be targeted.

### The structure of *pa*DXPS with thiamine analog provides evidence for the contribution of the diphosphate moiety in stabilizing a loop close to the active site

Thiamine analogs were previously reported as potent inhibitors of ThDP-dependent enzymes (26, 27). Several thiamine analogs had been synthesized in our group as inhibitors of DXPS (27, 28). We used one of these compounds to co-crystalize with our homologs as a diphosphate-free version of ThDP. The compounds were tested against *pa*DXPS and *kp*DXPS*.* The co-crystalized thiamine analog compound (2-{3-[(4-Amino-2-Methylpyrimidin-5-Yl)methyl]phenyl}ethanol) showed a half-maximal inhibitory concentration (IC_50_) of 197 μM for *pa*DXPS and 94 μM for *kp*DXPS. To further study the conformational changes in the active site loop, we set out to obtain an X-ray structure of either *pa*DXPS or *kp*DXPS with the thiamine analog. We were unsuccessful to generate cocrystals for *kp*DXPS with the inhibitor. We were, however, able to obtain a 2.2 Å co-crystal structure of *pa*DXPS (PDB ID: 8A4D).

**Figure 5:**
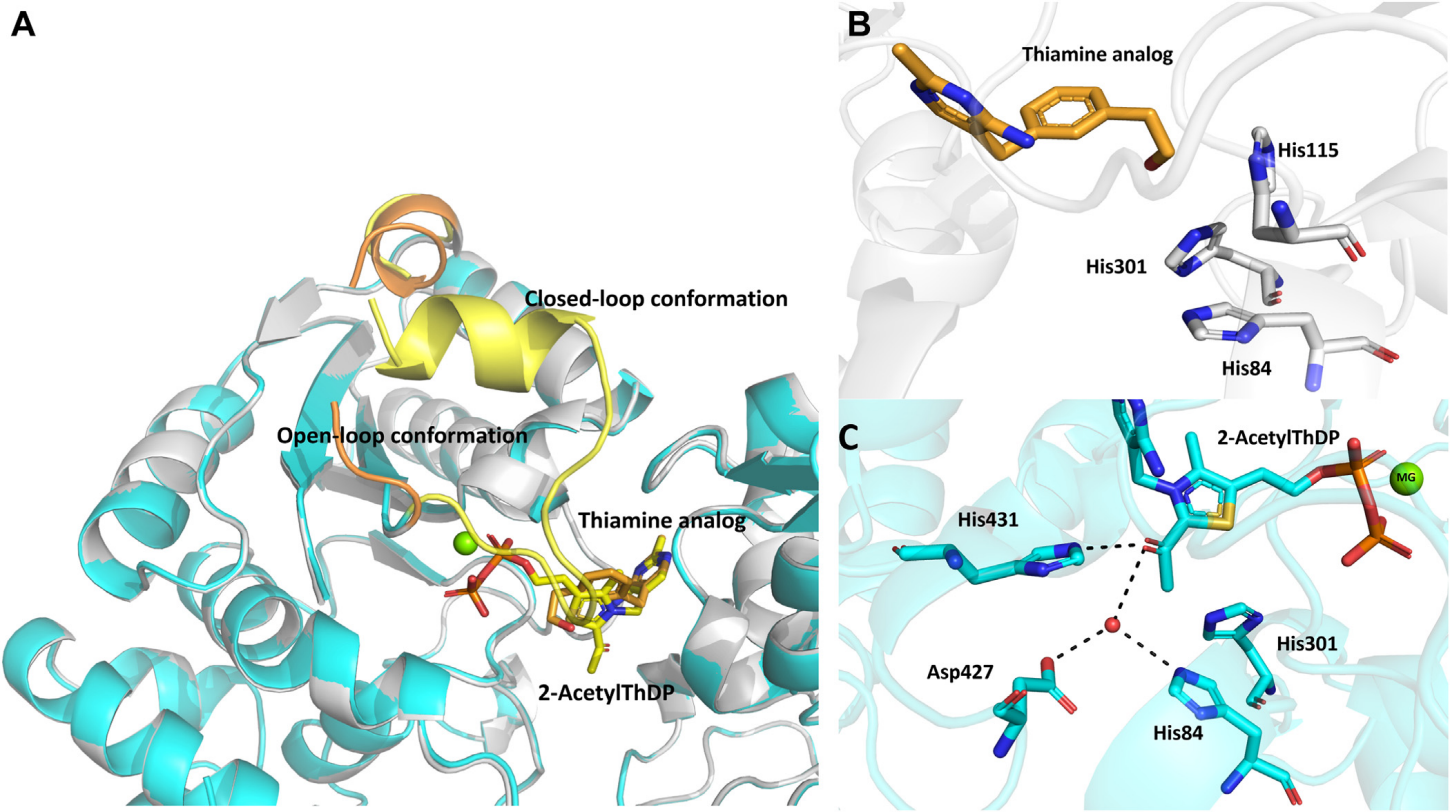
**Binding of the thiamine analog and 2-acetyl-ThDP with *pa*DXPS.***A*, thiamine analog bound structure in *gray* (loop in *orange*) and 2-acetyl ThDP structure in cyan (loop in *yellow*). *B*, zoomed in view of the Interactions between thiamine analog (*orange*) and binding residues of *pa*DXPS, the structure shows the hydroxyethyl part of the ligand pointing towards a histidine-rich region in the substrate binding site. The diphosphate loop shows an open conformation in the thiamine-bound structure, in *orange*. *C*, interactions between 2-acetyl-ThDP (cyan) and binding residues of *pa*DXPS, the structure shows a closed diphosphate loop conformation, in *yellow*. Interactions are shown as black, dashed lines (below 5 Å).

As anticipated, the binding of this analog is similar to the binding of the thiamine moiety of ThDP (pyrimidine and thiazole) with the pyrimidine ring stacking against Phe395. However, the phenyl ring is showing a slight deviation from the plane of thiazole binding seen with ThDP. The hydroxyl group of the ligand is pointing toward the entry site of the substrates while the flexibility of the hydroxyethyl group resulted in poor density for this part of the inhibitor. Nonetheless, it is surrounded by a histidine-rich region and shows the potential of H bonding to these residues (Fig. 5*B*). Compounds with shorter hydroxyl linkages show less activity (results not shown). Interestingly, the binding of this inhibitor excludes the part of Domain I that is involved in coordinating the diphosphate/Mg^+2^; the diphosphate loop shows the same open conformation as seen in the *apo* enzyme (Fig. 5*A*). This adds to the notion that this loop is stabilized by the interaction with this specific part of ThDP. This is an especially critical observation for drug discovery, as a different three-dimensional shape of the active site could enable the exploration of different regions of the DXPS in search for inhibitors.

### Structure of *pa*DXPS in the presence of the substrate analog fluoropyruvate shows the binding of acetylated ThDP and competitive inhibition

Fluoropyruvate is an analog of the natural substrate pyruvate; it was also described as the first inhibitor of DXPS from *P. aeruginosa* (9). DXPS decarboxylates pyruvate in an interaction mediated by ThDP in a stepwise manner: pyruvate first interacts with ThDP to form the intermediate lactyl ThDP (LThDP), this intermediate was shown to be stabilized by a histidine network near the C2 of ThDP. This reaction promotes a closed conformation of a loop close to the active site and the addition of D-GAP leads to a more open conformation, which eventually encourages decarboxylation (15, 17).

We performed an inhibition assay and a mode-of-inhibition (MOI) study on *pa*DXPS using fluoropyruvate. In the inhibition assay, fluoropyruvate inhibited the enzyme with an (IC_50_) value of around 27 μM. The MOI study indicated competitive inhibition with the natural substrate pyruvate (Table S3). Fluoropyruvate has also been reported to inactivate the E1 subunit of the pyruvate dehydrogenase complex, a mammalian ThDP-dependent enzyme. It was originally proposed that the fluoropyruvate interaction with the ThDP-bound enzyme results in a modification of a sulfhydryl group in the active site (8). Illustrated in Figure 1 is the interaction of fluoropyruvate with ThDP-bound *pa*DXPS as compared to pyruvate. To determine whether the inhibition of the enzyme is the result of the acetylation of a reactive residue in the active site after the addition of fluoropyruvate, we performed intact MS measurements to detect the acetylation of the enzyme by mass shift, intact mass results show similar masses for fluoropyruvate treated and untreated proteins (Fig. S5). These results, together with the MOI assay, suggest that no covalent modification is involved in inhibition by fluoropyruvate.

We then opted to get structural information on the fluoropyruvate-treated *pa*DXPS. The enzyme was incubated and crystallized with excess fluoropyruvate in the presence of ThDP and MgCl_2_. The structure was solved using molecular replacement and refined to 2.0 Å (PDB ID: 8A45). We found no evidence of an acetylated residue within the active site. On the other hand, we observed convincing electron density for the reaction intermediate 2-acetyl-ThDP in the active site (Fig. 5), consistent with the proposed reaction mechanism (Fig. 1). These findings indicate that inhibition with fluoropyruvate results rather from the acetylated ThDP, which is expected to be less reactive than ThDP and competes with the reactive enamine formed with pyruvate.

**Figure 6:**
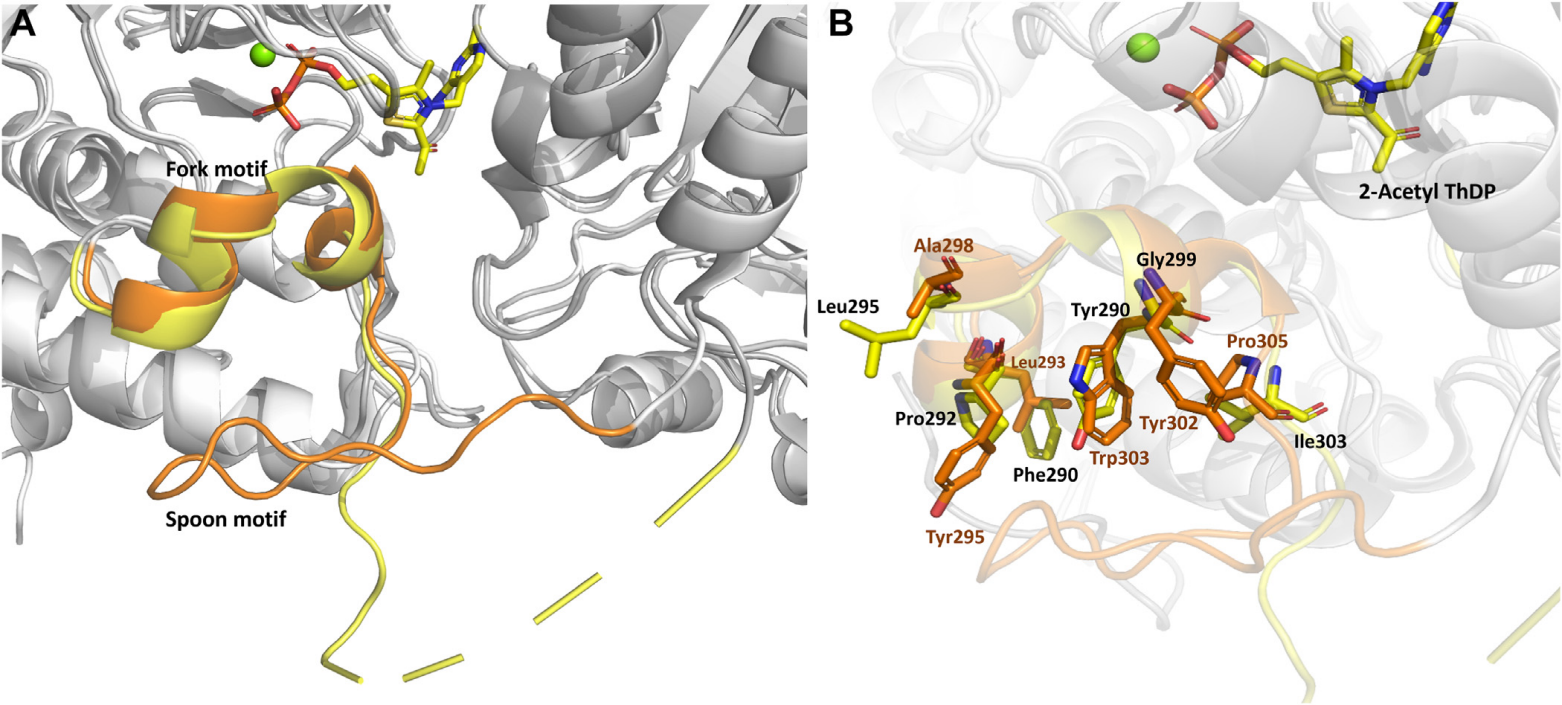
**Spoon-fork motif of *pa*DXPS.***A*, spoon-fork motif in *pa*DXPS in the presence of 2-acetyl THDP colored *yellow*, superimposed with *dr*DXPS in the presence of PLThDP, colored in *orange* showing the open conformation of the spoon motif in both structures. *B*, zoomed-in view of the fork motif highlighting changes in residues involved in stabilizing conformational changes in this region. Residues from *dr*DXPS are labeled in orange, from *pa*DXPS in *black*.

The structure of *pa*DXPS in the presence of fluoropyruvate provides a high-resolution view of acetylThDP-bound enzyme, a reaction product related to the enamine intermediate. The pyrimidine and diphosphate moiety of 2-acetyl-ThDP is bound in a highly similar way to ThDP (Fig. 5*A*), with the diphosphate loop also stabilized by coordinating residues Asp187, Asn216, and Met218 to the diphosphate/Mg part. Hydrogen bonds are formed between His-431 and the carbonyl of acetyl-ThDP. This histidine residue is conservatively preserved in other ThDP-dependent enzymes and has been shown to contribute to a relaxed/packed state, that ultimately drives decarboxylation (29). The conformation of this region is different in the structure of *mt*DXPS. *M. tuberculosis* replaces this interaction with a network of water-coordinated histidine, serine, and tyrosine (23). Interestingly, the carbonyl is also involved in coordinating water molecules with Asp427 and His84, while preserving the his431 conformation (Fig. 5*C*). The presence of these interactions results in a tetrahedral geometry around C2 of 2-acetylThDP, causing it to deviate from the plane of the thiazole, enabled by the single bond in the acetyl-ThDP compared to the double bond in the enamine. Our structure also shows the presence of His301, adding a positive charge to the pocket, which is missing in the structure with enamine in *dr*DXPS. This histidine is replaced by tyrosine in *ec*DXPS (14). His301 is also part of the spoon-fork motif, the flexibility of this site facilitates binding to the substrates and stabilizes intermediates (17). This region is significantly different from the DXPS structures. Figure 6 shows only some differences within the aligned fork motifs of the 2-acetyl ThDP *pa*DXPS structure and *dr*DXPS structure. In *kp*DXPS residues 246 to 284 are disordered and are also part of the spoon-fork motif. These differences might result in even more different conformational changes in this region during catalysis.

## Conclusion

This study introduces the first *apo* structures of DXPS from *P. aeruginosa* and *K. pneumoniae*, along with their cofactor ThDP-bound structures. These findings contribute to expanding our understanding of this important enzyme class and provide crucial structural insights for the advancement of innovative drug development targeting ESKAPE pathogens.

Upon ThDP binding, DXPS undergoes conformational changes that lead to an open and closed loop conformation near the active site. In the *pa*DXPS structure, the closed conformation is stabilized by interactions of the ThDP diphosphate/Mg^2+^ moiety with an asparagine-rich region of near the active site. In contrast, an open conformation is seen when this interaction is lost in the *apo* structure. This hypothesis was also confirmed by analyzing the crystal structure of *pa*DXPS with a thiamine analog lacking the diphosphate moiety, in which the same loop adopts the open conformation seen with the *apo* enzyme. The DXPS structure from *K. pneumonia* also shows loop movement deep in the active site, which leads to a much narrower pocket in the absence of ThDP.

From a drug-design perspective, these results open the door to different modes of DXPS inhibition. Our structures reveal the important role of the diphosphate moiety of ThDP in stabilizing part of the active site. Targeting specific residues in this loop may prevent the enzyme from binding to ThDP by not providing enough space for ThDP to bind, especially with the rearrangement of the loops in *kp*DXPS. In *pa*DXPS, one strategy could be to prevent the diphosphate-binding loop from closing, thereby preventing the enzyme from entering its active conformation. This could perhaps be achieved by using the pyrimidyl ring present in ThDP as a warhead and optimizing the rest of the compound to bind to the loop in its “open” conformation. Additionally, the binding mode of the thiamine analog indicates that it is also possible to explore compounds that target the substrate-binding channel in addition to the ThDP binding pocket. Following this approach, a three-headed inhibitor could perhaps be developed, which would likely show increased selectivity for DXPS over other ThDP-dependent enzymes due to the exploitation of DXPS’s unique binding-site geometry. Furthermore, we demonstrated that a competitive inhibition could be achieved by blocking further interactions of ThDP-bound enzyme with molecules that can form direct, less reactive intermediates with ThDP, therefore trapping the enzyme in this rate-determining kinetic step and preventing subsequent interactions with the natural substrates. These additional DXPS X-ray crystal structures from important ESKAPE pathogens lay the foundation for future structure-based drug design of potent and selective small-molecule inhibitors with a novel mode of action to treat bacterial infections.

## Experimental procedures

### Protein expression and purification

#### *pa*DXPS

The construct genes were synthesized commercially, cloned into pET28a, and transformed into BL21(DE3) for overexpression. The transformed cells were grown in a selective LB medium supplemented with Kanamycin (50 mg/ml) up to an OD600 of 0.6, then induced with 1 mM isopropyl-L-D-thiogalactoside (IPTG) and were subsequently grown at 18 °C for 16 h. The harvested cells were then disrupted in a microfluidizer after resuspension in a washing buffer consisting of 50 mM HEPES, 100 mM NaCl, 20 mM imidazole, and 2 mM β-mercaptoethanol (100 ml buffer per 25 g of wet cell pellet). After centrifugation, the supernatant was loaded onto a 5 ml HisTrap HP column equilibrated in washing buffer. After an extensive washing step with 30 column volumes of washing buffer, the bound fraction was then eluted with 50 mM HEPES, 100 mM NaCl, 300 mM Imidazole, and 2 mM β-mercaptoethanol. His-tag purification was followed by size-exclusion chromatography in 20 mM HEPES, 200 mM NaCl, and 2 mM β-mercaptoethanol. Sodium dodecyl sulfate-polyacrylamide gel electrophoresis (SDS-PAGE) and protein mass spectrometry were used to confirm the purity of protein purifications.

#### *kp*DXPS

The construct gene was synthesized commercially, cloned into pET28b+, and transformed into Lemo21(DE3) for overexpression. The transformed cells were grown in a selective LB medium at 37 °C supplemented with kanamycin (50 mg/ml) and chloramphenicol (34 mg/ml) up to an OD_600_ of 0.9, then induced with 0.1 mM isopropyl-L-D-thiogalactoside (IPTG), and were subsequently grown at 18 °C for 16 h. The harvested cells were then disrupted in a microfluidizer after resuspension in a washing buffer consisting of 20 mM Tris pH 8.0, 200 mM NaCl, 20 mM Imidazole pH 8.0, and 5% glycerol (100 ml buffer per 25 g of wet cell pellet). After centrifugation, the supernatant was loaded onto a 5 ml HisTrap HP column equilibrated in washing buffer. After an extensive washing step with 30 column volumes of washing buffer, the bound fraction was then eluted with an elution buffer consisting of 20 mM Tris pH 8.0, 200 mM NaCl, 250 mM imidazole pH 8.0, and 5% glycerol. Eluted protein was passed over a Hiprep 26/10 desalting column equilibrated in washing buffer to remove excess imidazole, after which TEV digestion was carried out by adding TEV protease in a 1:10 protein: TEV ratio and incubating the solution at 4 °C for 16 h. The N-terminal 6x His-Tag was removed by passing the protein over a 5 ml His-Trap HP column and collection of the flow-through. Purification was finalized by size-exclusion chromatography in 20 mM Tris, 200 mM NaCl, final pH 8.0 for *apo kp*DXPS and 10 mM HEPES, 200 mM NaCl, 5% glycerol, final pH 7.4 for ThDP-bound *kp*DXPS. SDS-PAGE and protein mass spectrometry were used to confirm the purity of protein purifications.

### Crystallization

#### pa*DXPS*

Crystallization trials were carried out with a protein solution containing 15 mg/ml *pa*DXPS in a storage buffer using commercially available screens from NeXtalbiotech. Ligand-bound structures were obtained after incubating *pa*DXPS with an excess amount of the ligand for 2 h at RT. In every case, crystals were grown in hanging drops with a reservoir solution consisting of 100 mM HEPES, 12% PEG-8000, and 200 mM calcium acetate. Plates were observed every 3 days and optimization of the identified conditions was carried out. 32% Glycerol was added as a cryo-protectant before the crystals were mounted in a cryo-loop and stored in liquid nitrogen until data collection.

#### kp*DXPS*

Crystallization trials were carried out with a protein solution containing 12.5 mg/ml *kp*DXPS (ligand-bound) and 47.5 mg/ml *kp*DXPS (*apo*) in the respective storage buffer using commercially available screens from NeXtalbiotech. Ligand-bound structures of *kp*DXPS were obtained by the addition of 1 mM ThDP/MgCl_2_ and incubating the solution for 4 h on ice before setting up the crystallization plates. For the protein complex, crystals were grown in sitting-drop optimization plates at 18 °C with a reservoir solution consisting of 100 mM HEPES pH 7.0 to 7.5 and 18 to 28% PEG-3000. For the *apo* version, crystals were grown in hanging-drop optimization plates at 18 °C with a reservoir solution consisting of 200 to 400 mM MgCl2, 100 mM HEPES pH 7.5, and 14 to 18% PEG-8000. In both cases, 15% 2*R*,3*R*-butanediol was added as a cryoprotectant before the crystals were mounted in a cryo-loop and stored in liquid nitrogen until data collection.

### Data collection and refinement

X-ray diffraction data for DXPS structures were collected at the Swiss Light Source located at the Paul Scherrer Institute in Switzerland. Specific information about each beamline can be found in the specific sections, all data collection and refinement statistics can be found in Table S2. Data reduction and scaling were done using AIMLESS (30) in CCP4i (31). Structures were solved using molecular replacement using the program Phaser.MR (32) in Phenix software (33). Repetitive cycles of refinement were done in COOT (34) and Phenix refine (35) to obtain final structures (Tables S1 and S2). Structures were validated using the Molprobity server and all figures were rendered using PyMoL.

### Kinetics and inhibition assays

To measure DXPS activity and kinetic parameters, a coupled spectrophotometric enzyme assay was adapted from the assay protocol described in the literature (36). A continuous kinetic photometric assay was used to measure DXPS activity. NADPH consumption by the auxiliary enzyme 1-deoxy-D-xylulose 5-phosphate reductoisomerase (IspC) was measured in a microplate reader (PHERAstar), monitoring the absorbance at 340 nm. In all cases (IspC) concentration was kept in excess (1 μM) to ensure DXPS is the only rate-limiting enzyme in the reaction mix. In the inhibition assays, in the case of the thiamine analog inhibitor, ThDP concentration was kept around 4X *K*_*m*_ (400 nM) to ensure the enzyme reaches maximum velocity while allowing competitive inhibition. In the case of fluoropyruvate, pyruvate concentration was also kept at 4X *K*_*m*_ (500 μM) for the same reason. For inhibition studies, DXPS activity was analyzed at RT for 30 min as follows; reaction mixture A (100 mM HEPES pH 7.5, 100 mM NaCl, 1 mM MgCl_2_, 0.5 μM NAPDH, 1 μM IspC, 400 nM ThDP, and 0.15 μM and 0.2 μM *pa*DXPS and *kp*DXPS respectively) was pre-incubated with different concentrations of inhibitor for 5 min at 37 °C in 10% DMSO. The reaction was then started by adding mixture B (100 mM HEPES pH 7.5, 100 mM NaCl, 2 mM DL-glyceraldehyde-3-phosphate and 2 mM pyruvate (thiamine analog inhibitor) and 500 μM pyruvate (fluoropyruvate). For kinetic assays, the cofactor ThDP and substrates pyruvate and D-GAP were used in varying concentrations. When a substrate or cofactor was kept constant, a concentration of 400 nM was used for ThDP and 2 mM for pyruvate and D-GAP. Blank correction and linear fitting of the absorption data were performed using the program Origin 2019 (OriginLab). The initial velocities obtained were plotted against the substrate concentrations, and the *K*_*m*_ values were determined by nonlinear curve fitting using the Michaelis–Menten model of the enzyme kinetics, Figs. S6 and S7.

## Data availability

Data supporting this study are included within the article and/or supporting materials.

## Supporting information

This article contains supporting information.

## Conflict of interest

The authors declare that they have no conflicts of interest with the contents of this article.
