# Supplementary material for: 1-deoxy-D-xylulose-5-phosphate synthase from Pseudomonas aeruginosa and Klebsiella pneumoniae reveals conformational changes upon cofactor binding
Source: J Biol Chem. 2023 Aug 9;299(9):105152. doi: 10.1016/j.jbc.2023.105152 (PMC10504544; doi:10.1016/j.jbc.2023.105152)
Supplement: Supporting Tables S1–S3 and Figures S1–S7 [file mmc1.docx]

**Supplementary:**

**1-deoxy-D-xylulose 5-phosphate synthase from *Pseudomonas aeruginosa* and *Klebsiella pneumoniae* reveals conformational changes upon cofactor binding**

***Rawia Hamid^1, 2^, Sebastian Adam^1^, Antoine Lacour^1^, Leticia Monjas^3^, Jesko Köhnke^4^, Anna K. H. Hirsch^1, 2*^.***

1. Helmholtz Institute for Pharmaceutical Research Saarland (HIPS) – Helmholtz Centre for Infection Research (HZI), Campus Building E8.1, 66123 Saarbrücken, Germany.

2. Department of Pharmacy, Saarland University, 66123 Saarbrücken, Germany.

3- Stratingh Institute for Chemistry, University of Groningen, Nijenborgh 7, NL-9747 AG Groningen, the Netherlands.

4- Institute of Food Chemistry, Leibniz University Hannover, Callinstr. 5 30167 Hannover, Germany. School of Chemistry, University of Glasgow, Glasgow  G12 8QQ, UK.

*Correspondence e-mail: [anna.hirsch@helmholtz-hips.de](mailto:anna.hirsch@helmholtz-hips.de)

| **Table S1**: Primary amino acid sequences of proteins used in this study. | **2** |
| --- | --- |
| **Table S2**: Data collection and refinement statistics | **3** |
| **Figure S1**: Sequence alignment of DXPS homologs | **4** |
| **Figure S2**: Native Ms analysis of *pa*DXPS | **5** |
| **Figure S3:** Calibration curve used to estimate molecular weights for DXPS |  |
| **Figure S4**: DXPS homologs active site comparison | **6** |
| **Figure S5:** Mass spectra of *pa*DXPS before and after incubation with fluoropyruvate | **7** |
| **Figure S6:** Michaelis-Menten kinetics analysis of *pa*DXPS and *kp*DXPS enzymes | **8** |
| **Figure S7:** Inhibitory dose-response curves IC50 of inhibitor on *pa*DXPS and *kp*DXPS | **9** |
| **Table S3**. Determination of MOI of fluoropyruvate against *pa*DXPS | **9** |

**Table S1**: Primary amino acid sequences of proteins used in this study.

| **Construct** | **Primary amino acid sequence** |
| --- | --- |
| Native paDXPS | MGSSHHHHHHSSGLVPRGSMENLYFQSHMPKTLHEIPRERPATPLLDRASSPAELRRLGEADLETLADELRQYLLYTVGQTGGHFGAGLGVVELTIALHYVFDTPDDRLVWDVGHQAYPHKILTERRELMGTLRQKNGLAAFPRRAESEYDTFGVGHSSTSISAALGMAIAARLQGKERKSVAVIGDGALTAGMAFEALNHASEVDADMLVILNDNDMSISHNVGGLSNYLAKILSSRTYSSMREGSKKVLSRLPGAWEIARRTEEYAKGMLVPGTLFEELGWNYIGPIDGHDLPTLVATLRNMRDMKGPQFLHVVTKKGKGFAPAELDPIGYHAITKLEAPGSAPKKTGGPKYSSVFGQWLCDMAAQDARLLGITPAMKEGSDLVAFSERYPERYFDVAIAEQHAVTLAAGMACEGMKPVVAIYSTFLQRAYDQLIHDVAVQHLDVLFAIDRAGLVGEDGPTHAGSFDISYLRCIPGMLVMTPSDEDELRKLLTTGYLFDGPAAVRYPRGSGPNHPIDPDLQPVEIGKGVVRRRGGRVALLVFGVQLAEAMKVAESLDATVVDMRFVKPLDEALVRELAGSHELLVTIEENAVMGGAGSAVGEFLASEGLEVPLLQLGLPDYYVEHAKPSEMLAECGLDAAGIEKAVRQRLDRQ |
| Mutated paDXPS | MGSSHHHHHHSSGLVPRGSMENLYFQSHMPKTLHEIPRERPATPLLDRASSPAELRRLGEADLETLADELRQYLLYTVGQTGGHFGAGLGVVELTIALHYVFDTPDDRLVWDVGHQAYPHKILTERRELMGTLRQKNGLAAFPRRAESEYDTFGVGHSSTSISAALGMAIAARLQGKERKSVAVIGDGALTAGMAFEALNHASEVDADMLVILNDNDMSISHNVGGLSNYLAKIGGGGGGPGTLFEELGWNYIGPIDGHDLPTLVATLRNMRDMKGPQFLHVVTKKGKGFAPAELDPIGYHAITKLEAPGSAPKKTGGPKYSSVFGQWLCDMAAQDARLLGITPAMKEGSDLVAFSERYPERYFDVAIAEQHAVTLAAGMACEGMKPVVAIYSTFLQRAYDQLIHDVAVQHLDVLFAIDRAGLVGEDGPTHAGSFDISYLRCIPGMLVMTPSDEDELRKLLTTGYLFDGPAAVRYPRGSGPNHPIDPDLQPVEIGKGVVRRRGGRVALLVFGVQLAEAMKVAESLDATVVDMRFVKPLDEALVRELAGSHELLVTIEENAVMGGAGSAVGEFLASEGLEVPLLQLGLPDYYVEHAKPSEMLAECGLDAAGIEKAVRQRLDRQ |
| Mutated kpDXPS | MSFDIAKYPTLALVDSTQELRLLPKESLPKLCDELRRYLLDSVSRSSGHFASGLGTVELTVALHYVYNTPFDRLIWDVGHQAYPHKILTGRRDKIGTIRQKGGLHPFPWRGESEYDVLSVGHSSTSISAGIGVAIAAAKEDKQRRAVCVIGDGAITAGMAFEAMNHAGDIKPDLLVVLNDNEMSISENVGALNNHLAGGGGGGGPGTLFEELGFNYIGPVDGHDVLGLVSTLKNMRDLKGPQFLHIMTKKGRGYEPAEKDPITFHAVPKFDHTSGVLPKSSGGLPSYSKIFGDWLCETAAKDNKLMAITPAMREGSGMVEFSKKFPDRYFDVAIAEQHAVTFAAGLAIGDYKPVVAIYSTFLQRAYDQVIHDVAIQKLPVLFAIDRAGIVGADGQTHQGAFDLSFLRCIPDMVVMTPSDENECRQMLYTGYHYSDGPCAVRYPRGSGTGATLEPLASLPIGKGVVKRQGEKIAILNFGTLLPEAAAVADKLNATLVDMRFVKPLDTALILQLAGEHDALVTLEENAIMGGAGSGVNEVLMAHRRAVPVLNIGLPDYFIPQGTQEEIRADLGLDAAGIEAKIRDWLA |

|  | Apo paDXPS | ThDP-bound paDXPS | paDXPS – 2-acetyl-ThDP | paDXPS – Thiamine analog | ThDP-bound kpDXPS | Apo kpDXPS |
| --- | --- | --- | --- | --- | --- | --- |
| PDB code | 8A29 | 8A5K | 8A45 | 8A4D | 8A9C | 8A8Y |
| Resolution range | 48.59–2.2 | 46.29–2.37 | 48.59–2.0 | 48.54–2.2 | 43.37 – 1.8 | 91.87 – 2.1 |
| Space group | P21 21 21 | P21 21 21 | P21 21 21 | P21 21 21 | P21 | C2 2 21 |
| Unit cell  a b c [Å]  α β γ [°] | 116.44 137.63 232.08  90.00 90.00 90.00 | 116.82Å 137.06Å 231.44  90 90 90 | 117.12 138.01 231.63  90.00 90.00 90.00 | 116.618 137.618 231.681  90.00 90.00 90.00 | 89.67 72.56 91.44  90.00 108.46 90.00 | 119.16 144.25 142.91  90.0 90.0 90.0 |
| Total reflections | 376678 (37313) | 325476 (32403) | 501348 (49688) | 371083 (36366) | 557529 (75397) | 323919 (45609) |
| Unique reflections | 188736 (18663) | 164247 (16316) | 187627 (18663) | 188257 (18546) | 100675 (14606) | 71565 (10344) |
| Multiplicity | 7.1 (7.3) | 2.0 (2.0) | 12.2 (12.3) | 4.5 (4.5) | 5.5 (5.2) | 4.5 (4.4) |
| Wavelength | 0.97 | 0.97 | 0.97 | 0.97 | 0.97 | 0.97 |
| Completeness (%) | 99.9 (100) | 99.46 (99.83) | 99.34 (99.50) | 99.63 | 97.9 (97.6) | 99.6 (99.6) |
| Mean I/ sigma (I) | 11.09 (2.65) | 6.52 (2.48) | 14.56 (3.47) | 6.86 (1.65) | 13.4 (2.5) | 9.9 (2.2) |
| Wilson B-factor | 19.66 | 25.62 | 19.67 | 25.24 | 25.41 | 35.38 |
| R-merge | 0.140 (0.953) | 0.07279 (0.3303) | 0.150 (1.187) | 0.1092 (0.5661) | 0.066 (0.561) | 0.089 (0.720) |
| R-work | 0.1896 | 0.1825 | 0.1948 | 0.1996 | 0.1593 | 0.1749 |
| R-free | 0.2254 | 0. 238 | 0.2261 | 0.2323 | 0.1902 | 0.1987 |
| Number of non-hydrogen atoms | 27762 | 27348 | 28232 | 28005 | 8837 | 7781 |
| Macromolecules | 25575 | 25645 | 25744 | 25672 | 7984 | 7384 |
| Ligands | 70 | 280 | 296 | 213 | 26 |  |
| Solvent | 2157 | 1527 | 2300 | 2222 | 827 | 397 |
| Protein residues | 3357 | 3370 | 3384 | 3366 | 1043 | 970 |
| RMS (bonds) | 0.003 | 0.019 | 0.005 | 0.002 | 0.006 | 0.003 |
| RMS (angles) | 0.63 | 0.93 | 0.70 | 0.55 | 0.79 | 0.56 |
| Average B-factor | 25.78 | 30.88 | 29.70 | 31.57 | 32.6 | 48.29 |
| Macromolecules | 26.85 | 30.86 | 29.30 | 29.40 | 31.84 | 48.42 |

**Table S2**: Data collection and refinement statistics of all X-ray structures shown in the manuscript. Statistics for the highest-resolution shell are shown in parentheses.

**Figure S1**: **Sequence alignment of DXPS homologs**: from *p. aeruginosa*, *K. pneumonia*, *d. radiodurans* and *E.coli*. The identity, shown as a bar graph above the sequences, was calculated using the four aligned sequences. Amino acids in the active site are indicated by red boxes. The truncated loop is underlined with bold blue line.

**Native Mass Spectrometry**Purified *pa*DXPS was buffer-exchanged into 10 mM ammonium acetate solution (pH 7.0) by repeated ultracentrifugation using 10K centrifugal filters. The protein was diluted to 5 µM to obtain the highest sensitivity in the mass spectrometer. Native MS analysis was conducted on a Bruker SolariX XR 7T Fourier-transform ion cyclotron resonance (FT-ICR) mass spectrometer equipped with a high-resolution electrospray ionization (HRESI) source (Bruker Daltonics, Billerica, MA, USA) by direct infusion of the mixture at a flow rate of 2 µL/min. Source parameters were set to 500V end-plate offset, 4000 V capillary voltage, 3 bar nebulizer gas pressure, 5 L/min dry gas, and 200 °C dry gas temperature. The instrument was calibrated using an Agilent ESI-L low-concentration tuning mix (G1969-85000). Profile spectra were recorded in positive ion mode with a mass range from 150 to 5000 m/z. Each spectrum was a sum of 128 transients composed of 512k data points. The pulse sequence control and data acquisition was controlled by ftms control software in a Windows operating system.


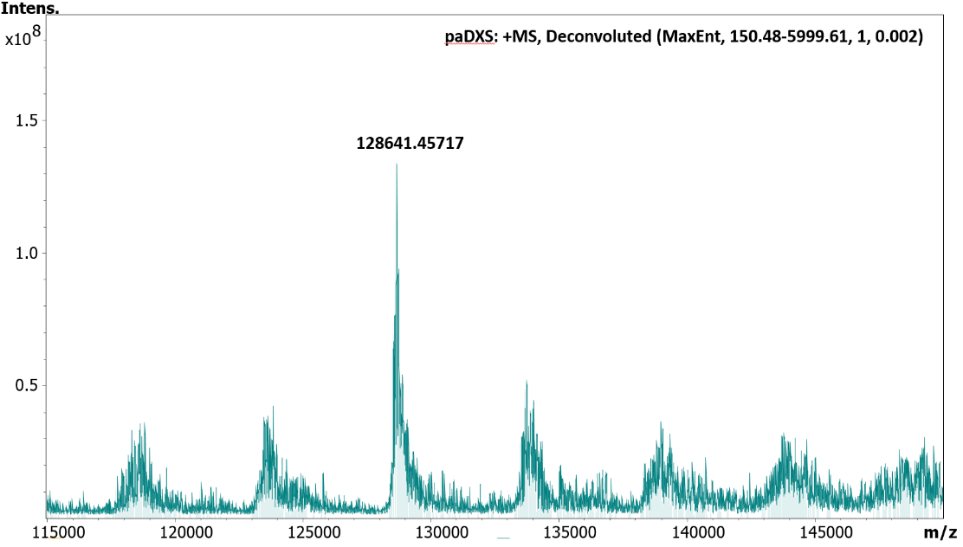


**Figure S2**: **Native Ms analysis of *pa*DXPS**. The full mass of the *pa*DXPS protein can be observed with a m/z of 128641 showing the presence of paDXPs as a dimer in solution, the mass corresponds to 2 paDXPS monomers (**~** 63965 Da), 2 ThDP units. The MS parameters on the Orbitrap EMR were optimized specifically for the m/z window of *pa*DXPS protein.

**
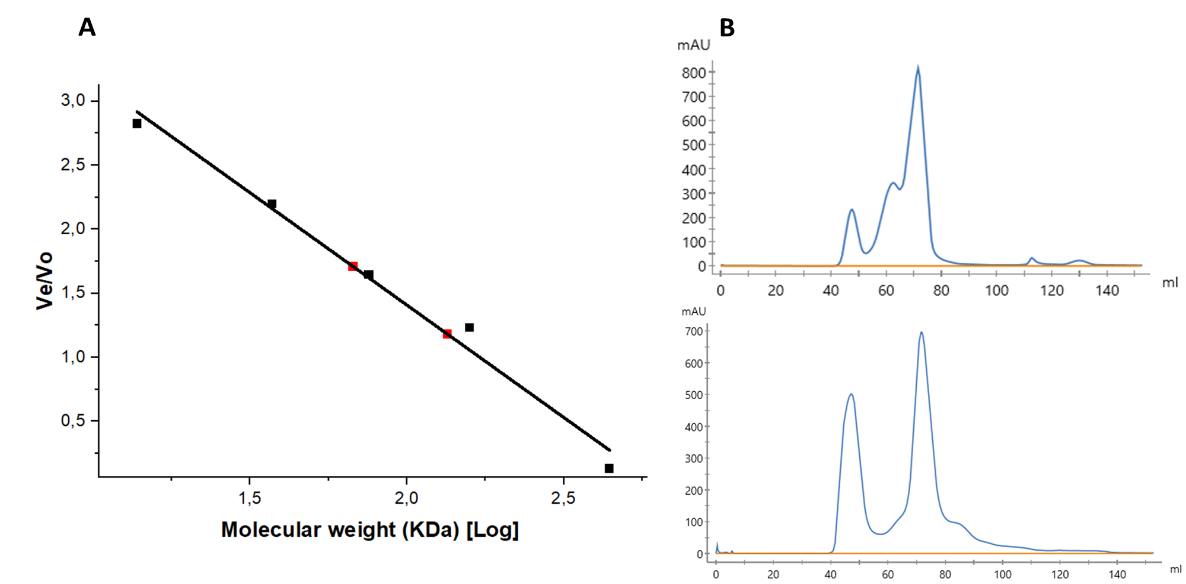
**

**Figure S3**: **A:** **Calibration curve used to estimate molecular weights for DXPS**. Size exclusion chromatography column S200 16/600 was calibrated using Thyroglobulin (670 KDa), y-Globulin (158 KDa), ovalbumin (44 kDa), Myoglobin (17 KDa), and Vitamin B12 (1,350 KDa). The calibration curve was plotted using the elution volume/ column void volume (Ve/V0) versus logarithm of the molecular weight. Straight line is the calibration curve calculated from the data for molecular weight standards. Red dots correspond to the positions of Ve/Vo values for DXPS. Linear equation, from the calibration curve was used to calculate the experimental molecular weights reported in this study. **B:** elution profile of kpDXPS showing elution in two peaks (UV spectra) corresponding to the monomer and the dimer in SEC.


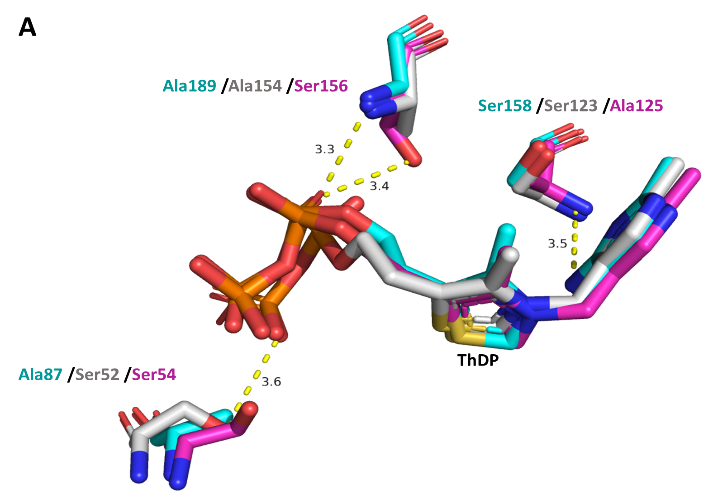


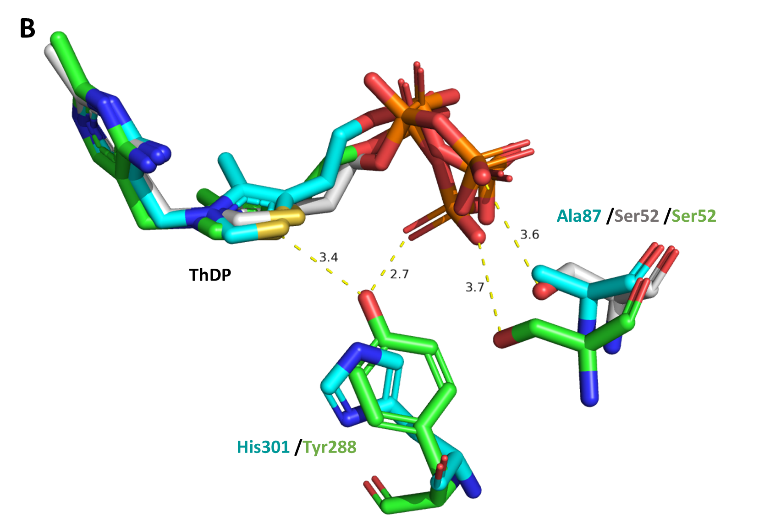


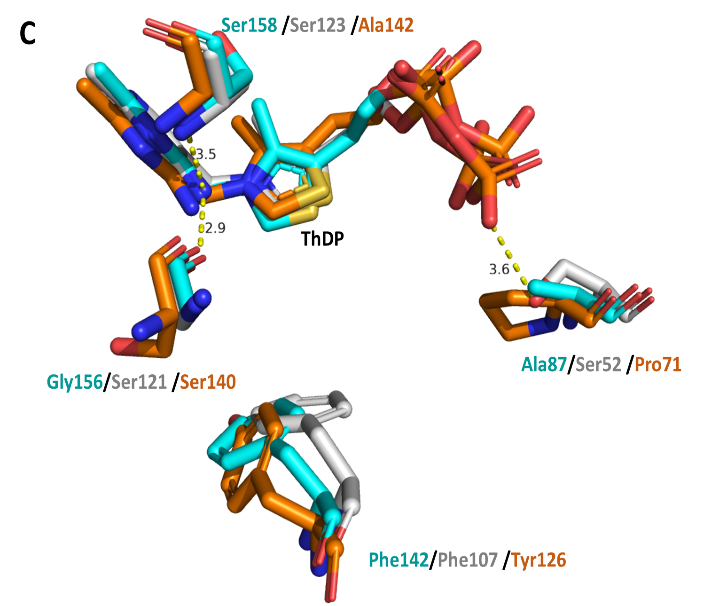


**Figure S 4: DXPS homologs active site comparison:** showing residues involved in binding to ThDP, highlighting differences in residues in/near the active site. **A** *pa*DXPS in cyan overlaid with *kp*DXPS in grey and the model homolog from *dr*DXPS in magenta, different residues between structures are labeled using the same color coding. **B** *pa*DXPS in cyan overlaid with *kp*DXPS in grey and *ec*DXPS in green. The difference in residues between structures is labeled using the same color coding. **C** *pa*DXPS in cyan overlaid with *kp*DXPS in grey and *mt*DXPS in orange, differences in residues between structures are labeled using the same color coding. Residue atoms are shown as sticks (C, cyan; O, red; N, blue; P, orange; S, yellow). Hydrogen bonds are shown as yellow dashed lines, and distances are calculated in Ångstrom.

**LC-MS spectroscopy:**

LC-MS measurements to determine the intact protein mass were performed using a Dionex Ultimate 3000 RSLC system equipped with an Aeris Widepore XB-c8 (150 x 2.1 mm, 3.6 µm particle diameter(dp)) column (Penomenex, USA). The LC was coupled to a maXis 4G high-resolution time-of-flight (HR-ToF) mass spectrometer (Bruker Daltonic, Germany) using an Apollo electrospray ionization (ESI) source. Separation of 5 µL protein sample on the LC was achieved by a linear gradient from solvent A (H2O plus 0.1% formic acid) to solvent B (acetonitrile plus 0.1% formic acid) as follows: 0 – 0.5 min (2%), 0.5 – 10.0 min (2 – 75%), 10.0 – 13.0 min (75%), 13.0 – 18.0 min (2%). The following conditions were used for mass spectrometry: capillary voltage 4,000 V, temperature 200 °C, dry gas flow rate 5 L / min and nebulizer 14.5 psi. Data were recorded in a mass range of 150 – 2,500 m/z. Sodium formate clusters were used for calibration of the maXis 4G spectrometer before every injection to avoid mass drift.

**Figure S5: Mass spectra of *pa*DXPS before and after incubation with fluoropyruvate.** No change in mass between treated and untreated protein in desaturated conditions indicates no covalent modification.

**Figure S6:** **Michaelis-Menten kinetics analysis, *K*_m_ values of paDXPS and kpDXPS enzymes**, analysis was conducted by varying pyruvate, D-GAP or ThDP concentration, enzyme concentration used: 150nM and 200 nM respectively. When a substrate or cofactor was kept constant, a concentration of 400nM was used for ThDP and 2 mM for pyruvate and D-GAP

**Table S3:** MOI of fluoropyruvate: IC_50_ values calculated with varying concentrations of pyruvate (0.8–10 fold *K*_m_), increasing in IC50 with increasing concentration of pyruvate indicating competitive inhibition. D-GAP and ThDP were kept at 2mM and 400nM in all measurements, respectively.

| [Pyruvate] (mM) | Fluoropyruvate IC50 (µM) |
| --- | --- |
| 4 | 154.9 ± 8.2 |
| 2 | 77.4 ± 9.8 |
| 1 | 42.6 ± 1.0 |
| 0.5 | 30.3 ± 1.3 |
| 0.25 | 27.0 ± 2.8 |
| 0.125 | 25.9 ± 1.3 |
| 0.0625 | 22.9 ± 3.2 |

**Figure S7:** Inhibitory dose-response curves to determine the IC_50_ for each inhibitor on *pa*DXPS and *kp*DXPS. The curves were fit using non-linear regression analysis program in OriginPro. In the case of the thiamine analog inhibitor ThDP concentration was kept at 4Xkm (400nM) to insure the enzyme reaches maximum velocity while allowing competitive inhibition. In the case of fluoropyruvate, pyruvate concentration was also kept at 4X km (500µM) for the same reason, 2mM D-Gap was used in both cases.
